# The hospital frailty risk score is of limited value in intensive care unit patients

**DOI:** 10.1186/s13054-019-2520-8

**Published:** 2019-07-02

**Authors:** Raphael Romano Bruno, Bernhard Wernly, Hans Flaatten, Fabian Schölzel, Malte Kelm, Christian Jung

**Affiliations:** 1 Division of Cardiology, Pulmonology, and Vascular Medicine, Medical Faculty, University Hospital Düsseldorf, Düsseldorf, Germany; 2 Clinic of Internal Medicine II, Department of Cardiology, Paracelsus Medical University of Salzburg, Salzburg, Austria; 3 Department of Anesthesia and Intensive Care, Haukeland University Hospital, Bergen, Norway; 4 Division of Gastroenterology, Hepatology and Infectiology, Medical Faculty, University Hospital Düsseldorf, Düsseldorf, Germany

**Keywords:** Frailty, Intensive care, Outcome mortality, Outcome non-mortality, Assessment

The identification of patients with frailty is of utmost importance, in particular during intensive care treatment of very old intensive care patients (VOPs). It is quite obvious that tools for this triage process should differ from younger patients. Frailty—not necessarily age—is associated with a negative impact on outcome, especially in critically ill patients [1]. This problem is of great importance as VOPs are one of the fastest growing subgroups in intensive care medicine. We expect an increase in the proportion of the world population older than 60 years from 12% in 2013 to 21% in 2050 [2].

Currently, there is an ongoing debate about which tool should be used for this purpose. In this context, we read with great interest about a novel ICD-10-code-based algorithm (hospital frailty risk score, HFRS) to identify frail patients at risk [3]. Until now, there has not been any field-testing for its value on the intensive care unit. Therefore, we performed a retrospective analysis and evaluated the impact of HFRS on outcome of ICU patients in our database containing 4381 ICU patients (described previously [4]). We included 1498 patients older than 75 years and calculated HFRS, APACHE-II, and SAPS-II scores for each patient individually. Survival rates were calculated using uni- and multivariable logistic regression intra-ICU mortality and both uni- and multivariable Cox regression analysis to adjust for confounding factors for the long-term combined endpoint of mortality and risk for readmission.

Table 1 demonstrates patients’ characteristics. As expected, survivors had significantly lower HFRS than non-survivors. HFRS was significantly associated with adverse outcome (HR 1.09 95%CI 1.05–1.13; *p* < 0.001). However, we found no independent association of HFRS after adjustment for APACHE-II scores (HR 1.03 95%CI 0.98–1.09 *p* = 0.27) or SAPS-II scores (HR 1.05 95%CI 1.99–1.11; *p* = 0.14) in a multivariable model.

**Table 1:** Baseline characteristics

	Survivors	Non-survivors	Total cohort	*P* value
HFRS	2.9 (± 3.3, *n* = 1259)	4.1 (± 3.5; *n* = 239)	3.1 (± 3.36; *n* = 1498)	< 0.001
Sex (male, [%])	60%	54%	59%	0.12
Age (mean, [years])	80.9 (± 4.2; *n* = 1259)	81.5 (± 4.33; *n* = 239)	81.0 (± 4.2; *n* = 1498)	0.07
Lactate [mmol/L]	2.0 (± 1.6; *n* = 1029)	6.1 (± 5.41; *n* = 184)	2.7 (± 3.0; *n* = 1213)	< 0.001
Creatinine [mmol/L]	149.4 (± 118.8; *n* = 1195)	206.9 (± 83.5; *n* = 229)	158.6 (± 122.1; *n* = 1424)	< 0.001
Urea [mmol/L]	12.9 (± 9.8; *n* = 1196)	17.7 (± 11.05; *n* = 228)	13.7 (± 10.2; *n* = 1424)	< 0.001
Albumin [g/L]	25.9 (± 6.2; *n* = 448)	21.1 (± 6.27; *n* = 103)	25.0 (± 6.5; *n* = 551)	< 0.001
Use of catecholamine	13%	18%	14%	0.12
Invasive ventilation	23%	59%	30%	< 0.001
Hemodialysis	8%	23%	11%	< 0.001

*HFRS* hospital frailty risk score. Normally distributed data points are expressed as mean ± standard deviation. Differences between independent groups were calculated using ANOVA. Categorical data are expressed as numbers (percentage)

This finding contrasts validating studies for the emergency department [5]. Possibly, there is a relevant lack in ICD coding for relevant comorbidities in very old patients on the ICU. In our field testing with realistic conditions in an ICU setting, HFRS does not independently predict risk in ICU patients above 75 years. In conclusion, frailty is complex and its detection crucial, but automatic electronic addition of ICD codes cannot replace the clinical assessment.

## Data Availability

The datasets used and/or analyzed during the current study are available from the corresponding author on reasonable request.
